# A Case of Successful Hybrid Treatment of Dual Arterial Bypass Using Indocyanine Green Fluorescence Angiography and Endovascular Treatment for Recurrent Superior Mesenteric Artery Aneurysm

**DOI:** 10.3400/avd.cr.23-00036

**Published:** 2024-01-12

**Authors:** Keisuke Yoshida, Yujiro Miura, Naoki Edo, Atsuyuki Mitsuishi, Tomohiro Matsumoto, Hiroyuki Kitagawa

**Affiliations:** 1 Department of Cardiovascular Surgery, Kochi Medical School Hospital, Nankoku, Kochi, Japan; 2 Department of Radiology, Kochi Medical School Hospital, Nankoku, Kochi, Japan; 3 Department of Surgery, Kochi Medical School Hospital, Nankoku, Kochi, Japan

**Keywords:** superior mesenteric artery aneurysm, hybrid therapy, indocyanine green fluorescence angiography

## Abstract

A 54-year-old woman with a mycotic superior mesenteric artery (SMA) aneurysm underwent emergent aneurysm resection with a great saphenous vein bypass. Follow-up computed tomography revealed a rapidly growing recurrent SMA aneurysm at the stump. Under the diagnosis of recurrent pseudoaneurysm of SMA with a fragile stump, we performed an open dual arterial bypass using indocyanine green fluorescence angiography and endovascular coil embolization. Subsequently, the patient’s recurrent mycotic SMA aneurysm was successfully managed without mesenteric ischemic complications. This method may help prevent fatal mesenteric ischemia during SMA aneurysm surgery.

## Introduction

Superior mesenteric artery (SMA) aneurysms account for 6.9% of all visceral artery aneurysms^1)^ and are typically found incidentally. However, some patients may experience nausea, pyrexia, and abdominal pain due to mesenteric ischemia. Rupture occurs in approximately 38% of SMA aneurysms at presentation, highlighting the importance of perioperative evaluation of arterial blood perfusion, including computed tomography (CT) angiography and intraoperative indocyanine green (ICG) angiography. These modalities help determine which mesenteric arteries require reconstruction and avoid bowel resection due to mesenteric ischemia.^2)^ This case study describes a patient with recurrent SMA aneurysm who required a hybrid of open bypass surgery using ICG angiography and endovascular treatment (EVT).

## Case Report

**Figure 1:**
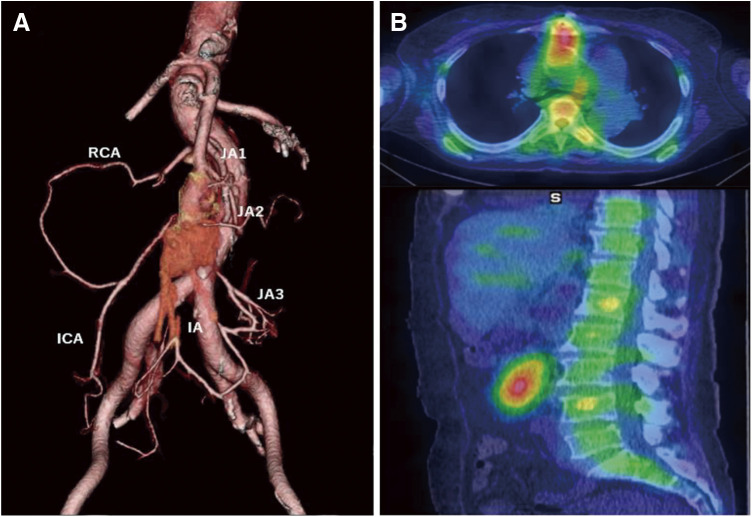
Fig. 1 (**A**) The SMA aneurysm was located 60 mm distal to the origin of the SMA and branched out as the third JA, the ICA, and IAs. The second JA and the right colic artery originated from the proximal neck of the SMA aneurysm. (**B**) Gallium citrate scintigraphy demonstrated an intense uptake in the prosthetic graft and SMA aneurysm. SMA: superior mesenteric artery; JA1: the first jejunal artery; JA2: the second jejunal artery; JA3: the third jejunal artery; RCA: right colic artery; ICA: ileocolic artery; IA: ileal artery

A 54-year-old woman with a history of aortic valve regurgitation (AR) and annuloaortic ectasia underwent valve-sparing aortic root and ascending aorta replacement two years prior. She had no personal or family history of connective tissue disorders. She presented to the emergency department 2 years after the initial surgery with epigastric pain, nausea, and decreased appetite. A CT angiography revealed a 13-mm SMA aneurysm located 60 mm distal to the origin of the SMA as well as splenic and renal infarctions. The SMA aneurysm branched into the third jejunal artery (JA), ileocolic artery (ICA), and ileal arteries (IAs), with obstructed IAs and observed collateral flow from the JAs (**Fig. 1A**). Blood cultures on admission were positive for *Staphylococcus epidermidis*. Gallium citrate scintigraphy demonstrated remarkably increased uptake around the prosthetic graft and SMA aneurysm (**Fig. 1B**). Transthoracic echocardiography revealed an increase in AR from mild to severe. She was diagnosed with mycotic aneurysms of the SMA secondary to infective endocarditis and thoracic prosthetic graft infection and was then referred to our institution.

**Figure 2:**
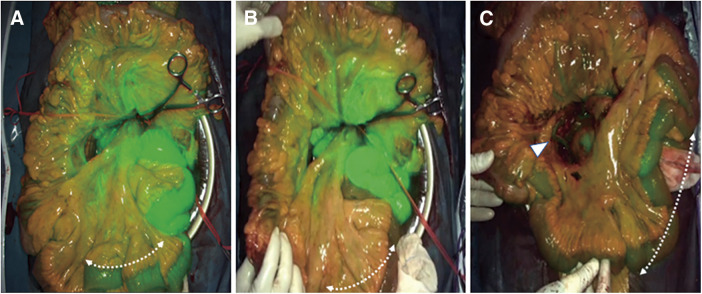
Fig. 2 (**A**) On ICG angiography, ICA fluorescence was observed before the ICA clamp test (double arrow). (**B**) After clamping ICA, ICA fluorescence was weak, without sufficient collateral flow (double arrow). (**C**) The GSV was anastomosed in a side-to-end fashion between the ICA and the right common iliac artery (white arrowhead). After reconstruction, ICA fluorescence was observed on ICG angiography (double arrow). ICG: indocyanine green; ICA: ileocolic artery; GSV: great saphenous vein

After the initiation of antibiotic therapy, the patient’s blood cultures became negative, and her symptoms resolved. However, she was readmitted for abdominal pain, and a CT scan 7 days after her admission showed a rapidly enlarging aneurysm measuring 23 mm, necessitating urgent open surgery. The proximal necks of the SMA aneurysm and ICA were dissected and clamped after systemic heparinization. ICG angiography revealed weak ICA fluorescence with insufficient collateral flow (**Figs. 2A** and **2B**); therefore, the bypass between the ICA and the right common iliac artery (CIA) was reconstructed using the great saphenous vein (GSV). Additionally, the proximal site of the ICA was suture-ligated. Following the reconstruction, ICA fluorescence was observed (**Fig. 2C**). The adhesions between the infectious SMA aneurysm wall and superior mesenteric vein (SMV) were severe; therefore, the mycotic aneurism was dissected as much as possible to prevent major complications. Seven days later, the patient suddenly developed a subarachnoid hemorrhage and underwent emergency coil embolization twice without sequelae. Thirty days after the abdominal surgery, the infected prosthetic graft was replaced with a rifampicin-bonded or soaked prosthetic graft during a bio-Bentall procedure. No microbes were detected in the culture studies of the extracted grafts and SMA aneurysm wall. She was discharged 6 weeks after antibiotic administration, and the infection remained adequately controlled with negative blood cultures.

**Figure 3:**
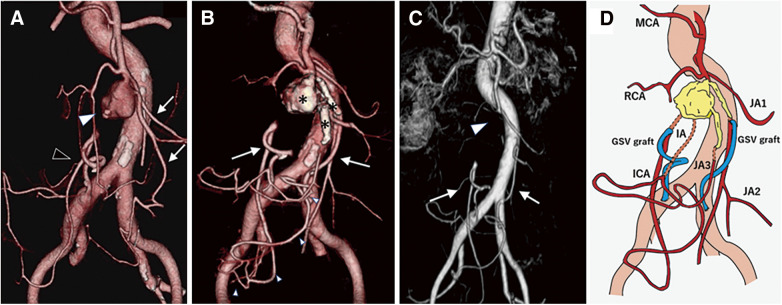
Fig. 3 (**A**) The recurrent SMA aneurysm had a diameter of 30 mm (white arrowhead) and branched out to form the second and third jejunal arteries (arrows). The GSV bypass graft was patent (black arrowhead). (**B**) Time-resolved magnetic resonance imaging demonstrated complete occlusion of the aneurysm (white arrowhead) and patency of all grafts (arrows). The SMA aneurysm and the second and third JAs were coil embolized (black stars). (**C**) All grafts were patent (arrows), and good collateral circulation was observed from the ICA to the third jejunal artery (JA3; white arrowhead). (**D**) The schematic diagram of **A**, **B**. SMA: superior mesenteric artery; MCA: middle colic artery; JA1: the first jejunal artery; JA2: the second jejunal artery; JA3: the third jejunal artery; RCA: right colic artery; ICA: ileocolic artery; IA: ileal artery; GSV: great saphenous vein

However, a follow-up CT 8 months later showed a recurrent SMA aneurysm measuring 30 mm, with the second JA proximal to the stump (**Fig. 3A**). Pseudoaneurysm formation due to a fragile stump was suspected. As securing the proximal site of the aneurysm without injuring the SMV was difficult, a hybrid approach was planned. First, the second and third JAs were identified and clamped, and ICG angiography revealed weak JAs fluorescence with insufficient collateral flow. Therefore, the GSV was harvested, and the left CIA was identified. An end-to-side anastomosis was performed between the second JA and the left CIA using the GSV graft, and the proximal segment of the second JA was ligated. After the second JA had been reconstructed, ICG angiography was performed to evaluate intestinal blood flow. The mesenteric marginal artery pulse was palpated, and peristalsis was confirmed before closing the abdomen. Following the open surgery, selective catheterization of the SMA was performed. Multiple coils from 4 to 24 mm were utilized to embolize the aneurysm, as well as the second and third JAs. Completion angiography demonstrated patency of the GSV grafts and marginal artery without aneurysm filling. The patient was discharged on postoperative day 12 and prescribed antiplatelet therapy. During the 6-month follow-up, time-resolved magnetic resonance angiography (TR-MRA) and CT angiography showed no recurrent perfusion of the aneurysm and patency of the dual bypass grafts (**Figs. 3B****–**3D****).

## Discussion

The treatment approach for SMA aneurysm depends on the location, size, and characteristics of the aneurysm, as well as the patient’s overall condition. Mycotic SMA aneurysm is typically managed through open surgery, where the affected arterial segment is resected and surrounding necrotic or infected tissues are debrided. EVT is preferred in cases where complete resection is not feasible, and the preoperative blood cultures yield negative results after antibiotic therapy.^3)^ However, patients who undergo EVT require careful follow-up owing to the higher risk of infection-related complications, reoperation, and readmission in the mid-term, compared to patients who underwent open surgery.^4)^ The hybrid approach, which combines surgery and endovascular techniques, is particularly useful in cases where EVT alone may cause mesenteric ischemia, while surgery alone may not achieve complete aneurysm resection.^5)^ In our case, debridement of the mycotic tissues was performed, and no microbes were detected in the culture studies of the extracted grafts and SMA aneurysm wall, indicating that the infection had been controlled by the previous surgery. Thus, a pseudoaneurysm was suspected because of the fragile arterial intima-media defect. In addition, collaboration between gastrointestinal surgeons and interventional radiologists is essential for the hybrid approach.

Both open surgery and EVT for SMA aneurysms involve sacrificing a branch of the SMA, which may cause mesenteric ischemia and significant morbidity if the collateral circulation from the celiac and inferior mesenteric arteries is inadequate. Therefore, mesenteric perfusion was evaluated using perioperative imaging. Currently, ICG angiography, transit-time flowmetry, X-ray angiography, CT angiography, and TR-MRA are the preferred methods to minimize complications. ICG angiography is an easy and safe method for the intraoperative assessment of intestinal perfusion, supporting real-time surgical decision-making.^2)^ ICG angiography is a qualitative evaluation tool and may not be sufficient for the assessment of intestinal congestion, and other methods can provide a comprehensive evaluation.^6)^ X-ray angiography is invasive but highly effective for institutional perfusion, and CT angiography facilitates the thorough evaluation of the patient’s anatomy. After coil embolization, it is difficult to assess the contrast enhancement of embolized lesions on CT owing to metallic artifacts. Alternatively, TR-MRA is a viable method for evaluating SMA aneurysms previously treated with embolotherapy.^7)^ Recently, attempts to measure and quantitatively evaluate the fluorescence brightness of ICG have been reported.^8)^ The limitation of this case is that we need further validation and clinical practice to establish the usefulness of ICG angiography for SMA aneurysm treatment, and the possibility of recurrence of dilatation cannot be denied in the future; thus, long-term follow-up is required.

## Conclusion

Our hybrid approach, which combines bypass surgery using ICG angiography and endovascular coil embolization, can successfully manage a case of recurrent SMA aneurysm. This method may help prevent fatal mesenteric ischemia during SMA aneurysm surgery.
